# Global Transcriptome Analysis in Influenza-Infected Mouse Lungs Reveals the Kinetics of Innate and Adaptive Host Immune Responses

**DOI:** 10.1371/journal.pone.0041169

**Published:** 2012-07-17

**Authors:** Claudia Pommerenke, Esther Wilk, Barkha Srivastava, Annika Schulze, Natalia Novoselova, Robert Geffers, Klaus Schughart

**Affiliations:** 1 Department of Infection Genetics, Helmholtz Centre for Infection Research and University of Veterinary Medicine Hannover, Braunschweig, Germany; 2 United Institute of Informatics Problems, National Academy of Sciences of Belarus, Minsk, Belarus; 3 Research Group Genome Analytics, Helmholtz Centre for Infection Research, Braunschweig, Germany; MRC National Institute for Medical Research, United Kingdom

## Abstract

An infection represents a highly dynamic process involving complex biological responses of the host at many levels. To describe such processes at a global level, we recorded gene expression changes in mouse lungs after a non-lethal infection with influenza A virus over a period of 60 days. Global analysis of the large data set identified distinct phases of the host response. The increase in interferon genes and up-regulation of a defined NK-specific gene set revealed the initiation of the early innate immune response phase. Subsequently, infiltration and activation of T and B cells could be observed by an augmentation of T and B cell specific signature gene expression. The changes in B cell gene expression and preceding chemokine subsets were associated with the formation of bronchus-associated lymphoid tissue. In addition, we compared the gene expression profiles from wild type mice with *Rag2* mutant mice. This analysis readily demonstrated that the deficiency in the T and B cell responses in *Rag2* mutants could be detected by changes in the global gene expression patterns of the whole lung. In conclusion, our comprehensive gene expression study describes for the first time the entire host response and its kinetics to an acute influenza A infection at the transcriptome level.

## Introduction

Influenza A virus has caused major pandemics in recent human history with millions of deaths. The most severe pandemic in 1918 resulted in about 30 million fatal casualties [1]. In addition, seasonal influenza infections represent a major health problem causing enormous losses of work force and deaths every year [2]. The course and outcome of an influenza A virus infection is influenced by several viral and host factors. It is thus important to understand the host response to an influenza infection in a more comprehensive fashion and to relate abnormalities of the phenotype with changes at the cellular and molecular level.

During an acute virus infection, highly dynamic and inter-related responses are triggered in the host at multiple levels which eventually result in clearance of the pathogen and establishment of a long-lasting immunity. In the early phase, infected cells and immune cells detect the presence of infectious microbes via membrane-associated and intracellular pathogen recognition receptors (PRRs). Activation of PRRs results in the stimulation of signaling pathways that lead to transcription of early response genes, mainly interferons, chemokines and cytokines (reviewed in [3], [4], [5], [6], [7], [8], [9]). Chemokines and cytokines are induced in the infected tissue and activate resident immune cells, mainly macrophages and dendritic cells (DCs), resulting in a coordinated and sustained chemokine/cytokine production and attracting infiltrating cells of the innate immune system, such as macrophages, granulocytes, NK cells, and DCs (reviewed in [4], [6], [7], [10]). The importance of NK cells for the host response has been confirmed in mice knock-out mutants in which the *Ncr1* gene has been deleted [11]. DCs take up antigens by direct infection or phagocytosis of infected dead cells, migrate to the draining lymph nodes where they activate T cells and present pathogen-specific antigens to them (reviewed in [7], [10]). This process causes the generation of antigen-specific T cells and the production of neutralizing antibodies (reviewed in [7], [10], [12], [13]). Finally, through the combined action of innate and adaptive immune responses, the infectious pathogen becomes inactivated and cleared from the body, repair processes start to resolve the tissue damages and long-term immunity will be established, including the formation of local bronchus-associated lymphoid tissues (reviewed in [7]).

Many individual aspects of these host-pathogen interactions during the course of an influenza infection have been studied. However, the dynamic changes over the entire time course of an infection were not described yet in a single experimental setting but rather individual aspects were studied with varying circumstances, experimental conditions and for limited time periods.

**Figure 1:**
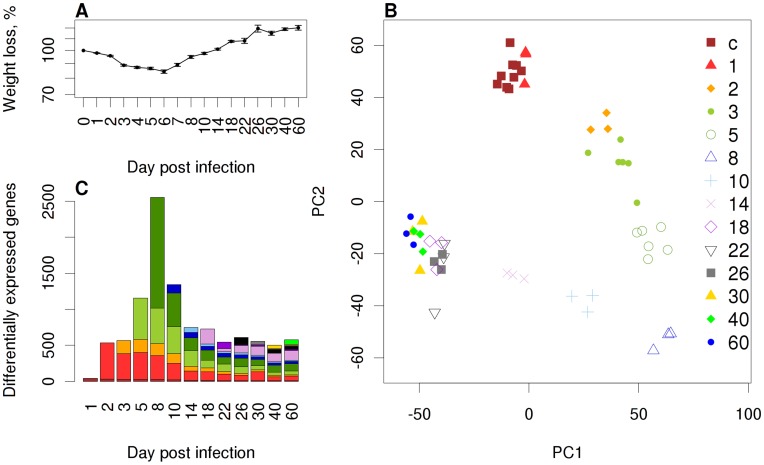
Global analysis of gene expression changes in the lungs of influenza infected C57BL/6J mice. (A) Weight loss of PR8M-infected C57BL/6J mice over two months after infection showing mean values +/− SEM. (B) A Principle Component Analysis (PCA) of all samples taken over the investigated time interval on the basis of the expression of all genes was conducted and single replicates were plotted with reference to the first two principal components (PC1, PC2) covering 57.3% of the variance. Symbols and colors indicate biological replicate samples prepared at the same day p.i. from different individuals. (C) A total of 3,595 differentially expressed (DE) genes were identified over the course of two months after infection. Bars indicate the number of DE genes on each day p.i. Colors indicate the number of DE genes that were newly detected at any given day, *e.g.* dark red: DE genes newly identified on day 1 p.i. compared to controls; dark green: DE genes that appeared at day 8 p.i. and were not differentially expressed at any time before. y-axis: total number of DE genes, x-axis: day after infection.

Here, we performed a systematic study by determining the global changes in gene expression patterns in the lungs over a period of 60 days in mice infected with influenza A virus, thereby capturing the dynamics of the system and relating changes in the transcriptome to immunological processes at the cellular and organ level. Our studies revealed that the different phases of the host response, such as infiltration of NK, T and B cells are well reflected by the changes in the transcriptome. Furthermore, analysis of gene expression changes in *Rag2* mutant mice after infection and in comparison to wild type expression profiles clearly highlighted the deficiency in the T and B cell response in *Rag2* mutant mice and verified the specificity and sensitivity of our analyses. In conclusion, our transcriptome analysis will provide an important basis for future systems biology studies to investigate and model host-pathogen interactions during an acute viral infection.

## Results

### Global Transcriptome Analysis of the Lung distinguishes Different Phases of the Host Response after Influenza Virus Infection

Wild type C57BL/6J mice were infected with the mouse-adapted influenza A virus PR8 (H1N1) and genome-wide gene expression patterns were analyzed for 60 days post infection (p.i.; Figure 1). During this time period, infected mice lost weight until day 6–8 p.i. and subsequently started to regain body weight and the initial weight was resumed at about 14 days p.i. (Figure 1A). Lungs were collected from infected mice and gene expression profiling was performed for whole lungs using microarrays. A principal component analysis (PCA) of all preprocessed probe data showed a temporal progression of global expression changes over time and a high reproducibility of measurements between biological replicates (Fig. 1B). The transcriptomes from all infected lungs were different from mock-infected controls, and the strongest difference to mock-infected mice was observed at day 8 p.i. However, from day 18 to day 60 p.i., minimal changes were observed over time but they still differed from mock-infected controls (see also below, *Expression of late host response genes indicates lung repair processes*). These observations are also reflected in the quantitative changes of differentially expressed (DE) genes (Figure 1C). The number of DE genes peaked at day 8 p.i. and after two months p.i., more than 500 genes were still differentially expressed compared to mock-infected controls (Table S1). Hence, on a global scale, considerable quantitative and qualitative changes in the gene expression patterns were observed in infected but not in control mice lungs until day 60 p.i.

**Figure 2:**
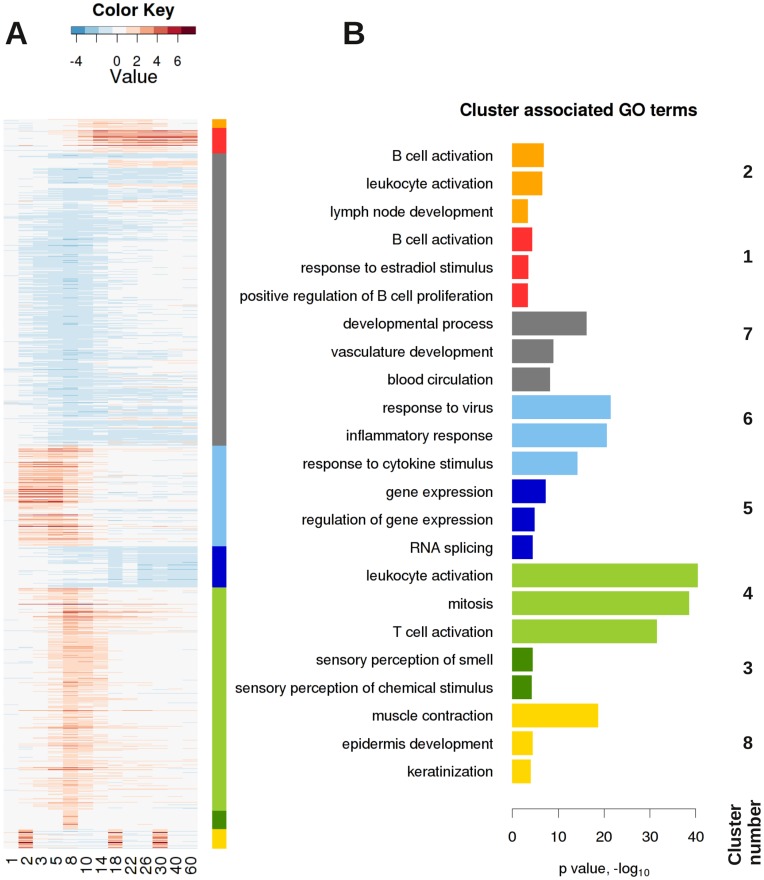
Density clustering of expression changes of DE genes in the lungs of influenza infected C57BL/6J mice. (A) In total, eight different groups of DE genes with similar expression kinetics were identified by cluster analysis as shown in the heat map. (B) The genes for the individual clusters were mapped to significantly associated GO terms of which the most representative terms are depicted. Genes in four clusters were up-regulated and functionally associated with immune responses: Cluster 6 represented a set of 445 DE genes and GO term analysis revealed cytokine production as the most dominating term. Cluster 4 (light green) contained 988 DE genes which were associated with T cell as well as apoptosis-related GO terms. Cluster 2 (orange) contained 37 and cluster 1 (red) 114 genes, respectively, and both were connected to B cell activation. The member genes of each cluster and the results of the GO term analysis are presented in Table S1 and Table S2).

To determine groups of genes with similar expression profiles, we performed a density cluster analysis of all DE genes over the time course of infection. In total, eight clusters were discernible (Figure 2, Table S1). Four clusters, exhibiting a unique temporal profile and being associated with immune responses, were further characterized. Cluster 6 contained 445 genes that were activated in the early phase of the host response, with a peak at day 2–5 p.i. and then down-regulated from day 5 p.i. on (Figure 2, cluster 6). The 50 most strongly expressed genes represented members of the chemokine/cytokine network (*e.g. Cxcl10*, *Ccl2*, *Ccl7*) and interferon activated genes (*e.g. Ifi205*, *Irf7*). Subsequent GO term statistical analysis of genes in this cluster confirmed that cytokine and chemokine production and response to virus were significantly over-represented (Figure 2 and Table S2). Cluster 4 contained genes that were stimulated during the intermediate phase of the host response with a peak at day 8 p.i. encompassing 998 DE genes (Figure 2, cluster 4). GO-term analysis and the 50 most strongly expressed genes (*e.g.Cd8b1, Cd3d, Gzmb,* Cxcl9) indicated that this cluster comprised mainly genes involved in T cell activation as well as apoptosis induction (Figure 2 and Table S2). Interestingly, genes of cluster 1 and 2 were activated during the late phase of the host response. They both demonstrated a steady increase from day 3–5 p.i. on and gradual decline after 14 days p.i., however, this descent was weaker in cluster 1. The 37 genes in cluster 2 (*e.g. Spib, Bcl11a, Cd22, Cd19*) indicated B-cell specific genes and genes involved in B cell activation by GO analysis (Figure 2 and Table S2). Similarly, cluster 1 contained 114 genes (e.g. *Ighg, Ighm*) was characterized by GO terms like B cell activation.

To further investigate the activation of specific gene regulatory pathways, we performed another global test, the signaling pathway impact analysis (SPIA). SPIA considers both the network topology of KEGG pathways and the signed fold change expression. The RIG-I pathogen sensory pathway (Figure S1A) as well as the chemokine/cytokine genes and receptors (Figures S1B, C) of the early viral host response were readily detectable early after infection. Most notably, the peak and the decline of the chemokine/cytokine genes and receptors coincided with the presence of HA transcripts and infectious viral particles in infected lungs [14]. In addition, NK and T cell pathways were found to be activated with a peak at day 8 p.i. (Figure S1D, E). The B cell receptor pathway appeared significantly regulated after day 5 p.i. (Figure S1F).

Taken together, at the global scale, clustering/GO term analysis and SPIA revealed an early innate immune response phase which increased rapidly within the first two days p.i. and then declined, followed by the activation of T cell pathways which peaked at about day 8 p.i. and a late B cell activation - solely based on transcriptome data.

### Activation of Distinct Interferon Responses during the Early and Intermediate Phase

One of the most important host responses to viral infections is the expression of interferons (IFNs). Three types of IFNs are generally distinguished: type I (IFN.

**Figure 3:**
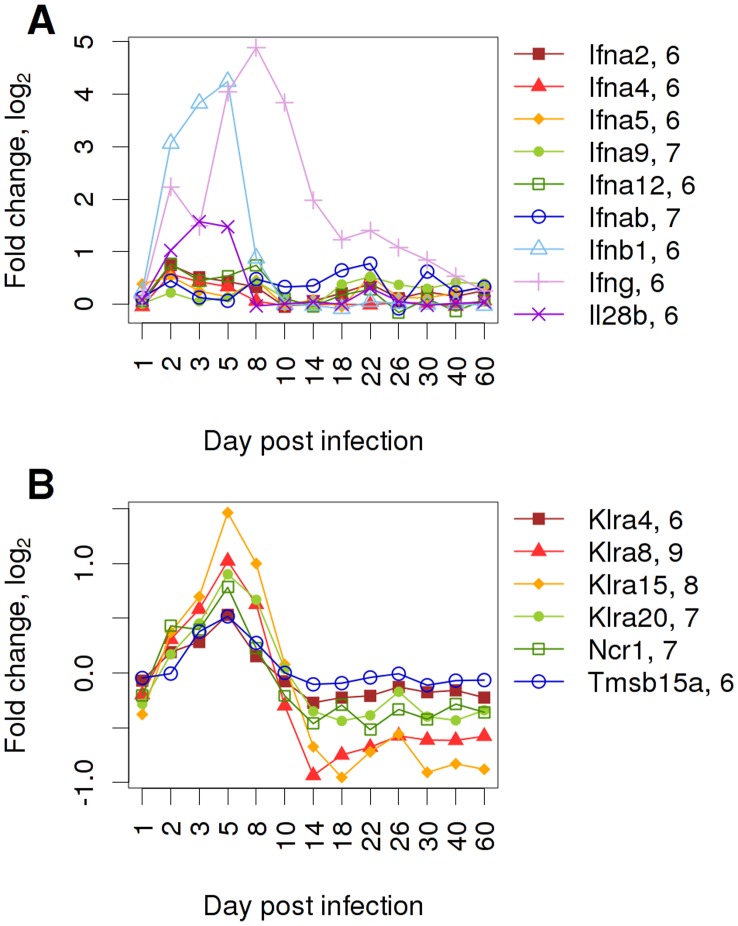
Activation of innate immune responses in C57BL/6J mice after influenza infection. Expression changes of (A) interferon type I, II, and III and (B) natural killer cell signature genes. Expression changes were represented as differences to the expression levels in mock-infected mice on a log_2_ scale. Please note that in this figure all interferon genes are depicted, even if they did not exhibit a more than two-fold increase of gene expression during the course of infection. The numbers after the gene names indicate the initial log_2_ expression values mock-infected control mice at day 2, 4, 14 after treatment. Gene names: interferon alpha 2 (*Ifna2*); interferon alpha 4 (*Ifna4)*; interferon alpha 5 (*Ifna5)*; interferon alpha 9 (*Ifna9)*; interferon alpha 12 (*Ifna12)*; interferon alpha B (*Ifnab)*; interferon beta 1, fibroblast (*Ifnb1*); interferon gamma (*Ifng*); interleukin 28b, interferon lambda (*Il28b*); killer cell lectin-like receptor, subfamily A, member 4 (*Klra4*); killer cell lectin-like receptor, subfamily A, member 8 (*Klra8*); killer cell lectin-like receptor, subfamily A, member 15 (*Klra15*); killer cell lectin-like receptor subfamily A, member 20 (*Klra20*); natural cytotoxicity triggering receptor 1 (*Ncr1*); thymosin beta 15a (*Tmsb15a*).

α and IFN β), type II (IFN γ) and type III (IFN λ). Up-regulation of type I and III IFNs was clearly seen during the early phase of infection by a strong increase in the expression of *Ifnb1*, a moderate augmentation of *Il28b* (IFN λ) and a slight but distinct accession in several *Ifna* genes (Figure 3A). The up-regulation of these early effector molecules paralleled the activation of the RIG-I-like receptor signaling pathway (Figure S1A). The expression of *Ifng* displayed a different kinetics. Its expression was activated early but continued to increase after day 5, reached a peak at day 8 and was still elevated at day 14 whereas the type I and III interferons were declining rapidly after day 5. These expression profiles are consistent with the general notion that type I and III interferons are mainly produced by infected epithelial cells and activated DCs in the early phase, and that *Ifng* is mainly expressed in activated and infiltrating NK and T cells in the early and intermediate phase of an infection.

### Expression Changes in Signature Genes Reveal the Activation and Infiltration of NK Cells during the Early and Intermediate Phase

NK cells play a crucial role in the defense of an influenza virus infection. Therefore, we defined a set of signature genes to follow the activation and infiltration of NK cells in infected lungs. For this, we purified NK and T cells from infected lungs at 3 days p.i. and performed gene expression profiling on the two cell populations. Genes that exhibited a high level of expression in the NK cell data set compared to the whole lung and which were not found to be expressed in T cells were selected as NK cell specific signature genes (data not shown). These signature genes increased in infected lungs shortly after infection and exhibited a peak at day 5 p.i. (Figure 3B). In conclusion, the up-regulation of NK cell signature genes was readily detectable at the early, innate immune response phase and continued into the intermediate phase of the host response.

### Up-regulation of T-cell Signature Genes Reveals the Infiltration of T Cells during the Intermediate Phase of Infection

**Figure 4:**
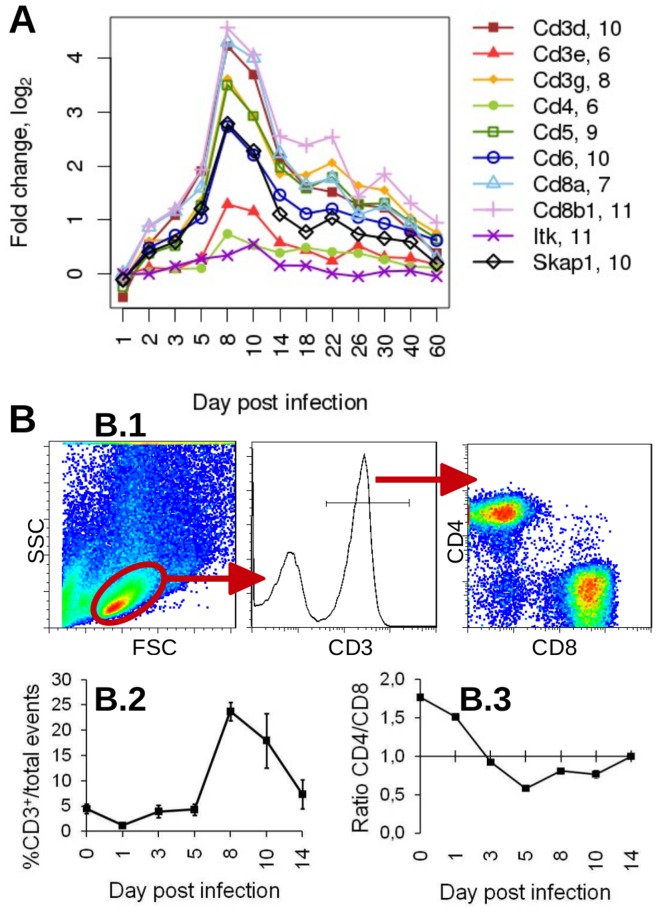
Kinetics of expression changes for T cell signature genes after influenza infection of C57BL/6J mice and corresponding T cell infiltration. (A) Gene expression pattern of T cell signature genes showing the changes in expression levels compared to mock-infected mice (Spearman’s rho ≥0.76 except for *Itk*). Numbers behind the gene names indicate the initial log2-expression values mock-infected control mice at day 2, 4, 14 after treatment. (B) Dynamics of CD3+ T cell infiltration into infected lungs analyzed by flow cytometry (day 0: non-infected controls). Please note that in this figure all signature genes are depicted, even if they did not exhibit a more than two-fold increase of gene expression during infection. (B.1) Forward (FSC) and sideward scatter (SSC) plot indicating the signals which were selected for further analysis of CD3^+^ T cells and CD4 and CD8 expression. (B.2) Kinetics of CD3^+^ cells in lung homogenates calculated as percentage of total events. The mean of three values and the respective SEM are depicted. One representative of two independent experiments is shown. (B.3) Ratio of CD4/CD8 cells within the CD3^+^ T cell population. Gene names: CD3 antigen, delta polypeptide (*Cd3d*); CD3 antigen, epsilon polypeptide (*Cd3e*); CD3 antigen, gamma polypeptide (*Cd3g*); CD4 antigen (Cd4); CD5 antigen (*Cd5*); CD6 antigen (*Cd6*); CD8 antigen, alpha chain (*Cd8a*); CD8 antigen, beta chain 1 (*Cd8b1*); IL2-inducible T-cell kinase (*Itk*); src family associated phosphoprotein 1 (*Skap1*).

To follow the activation and infiltration of T cells in the lung, we investigated the changes in expression levels of T cell signature genes. These signature genes were defined previously by our group from gene expression patterns that were generated from lung transcriptomes of non-infected BXD recombinant inbred strains. In this data set, we identified a group of T cell genes which was characterized by an expression profile that strongly correlated with the T cell marker *Cd3d* and which was highly specific for T cells with reference to the BioGPS database [15]. After infection, a strong augmentation in the expression of the T cell signature genes was observed starting at day 5, with a peak at day 8, and followed by a decline (Figure 4A). This kinetic corresponded to the changes observed at the intermediate phase of the host response both by the cluster analysis (cluster 4, Figure 2) and by the SPIA results (Figure S1E). The time period of T cell infiltration into the lung at day 8 p.i. could be confirmed experimentally by flow cytometry (Figure 4B). Furthermore, a stronger expression signal was observed for *Cd8* compared to *Cd4* at day 8 p.i. which was also consistent with an increase in the CD4/CD8 cell ratio detected by flow cytometric methods (Figure 4B.3).

Hence, the expression changes of the T cell signature genes in the lung transcriptome corresponded very well with the infiltration and activation of T cells and can thus serve as *bona fide* marker set to follow this branch of the adaptive immune response.

### Increase in Expression of B Cell Specific Signature Genes is Associated with the Appearance of BALT in the Lung during the Late Phase of Infection

**Figure 5:**
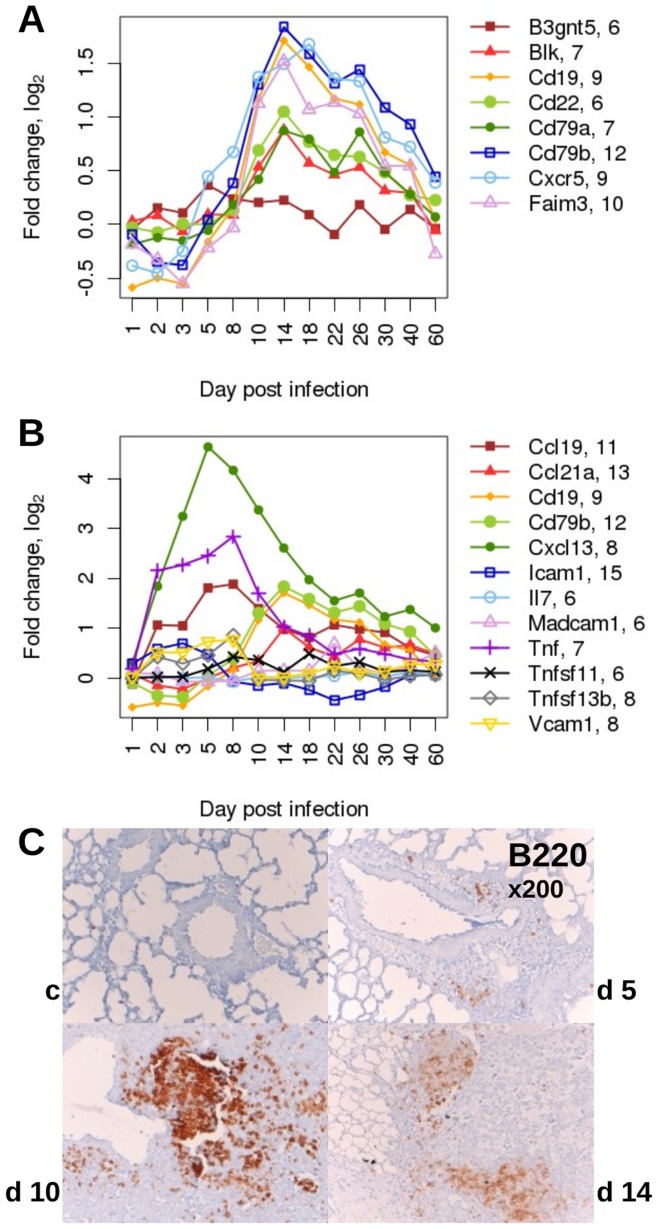
Kinetics of expression changes for B cell signature genes and analogue appearance of B cells in newly induced bronchus-associated lymphoid tissue (BALT) after influenza infection of C57BL/6J mice. (A) Gene expression changes of B cell signature genes compared to mock-infected mice (Spearman’s rho ≥0.85 except for *B3gtn5*). (B) Expression of signaling molecules associated with induction of secondary lymphoid organs precedes BALT formation in the lung. Values are given in expression fold changes compared to mock-infected mice. Numbers behind the gene names indicate the initial log_2_ expression values mock-infected control mice at day 2, 4, 14 after treatment. Please note that in this figure all signature genes are depicted, even if they did not exhibit a more than two-fold increase of gene expression during infection. (C) Histological sections stained for expression of B220 indicating the newly formed BALT at days 5, 10 and 14 p.i. Gene names: UDP-GlcNAc:betaGal beta-1,3-N-acetylglucosaminyltransferase 5 (*B3gnt5*); B lymphoid kinase (*Blk*); CD19 antigen (*Cd19*); CD22 antigen (*Cd22*); CD79A antigen (immunoglobulin-associated alpha) (*Cd79a*); CD79B antigen (*Cd79b*); chemokine (C-X-C motif) receptor 5 (*Cxcr5*); Fas apoptotic inhibitory molecule 3 (*Faim3*); chemokine (C-C motif) ligand 19 (*Ccl19*); chemokine (C-C motif) ligand 21A (serine) (*Ccl21a*); chemokine (C-X-C motif) ligand 13 (*Cxcl13*); intercellular adhesion molecule 1 (*Icam1*); interleukin 7 (*Il7*); mucosal vascular addressin cell adhesion molecule 1 (*Madcam1*); tumor necrosis factor (*Tnf*); tumor necrosis factor (ligand) superfamily, member 11 (*Tnfsf11*); tumor necrosis factor (ligand) superfamily, member 13b (*Tnfsf13b*); vascular cell adhesion molecule 1 (*Vcam1*).

Cluster analysis and SPIA indicated that B cells were recruited into the lung during the late phase of the host response (Figures 2, S1F). Therefore, we also followed the expression patterns of B cell signature genes after infection. The B cell-specific signature genes were obtained from our previous BXD lung expression data set by genes strongly correlating to the expression of the B cell marker *Cd19* and exhibiting a B cell restricted expression profile in the BioGPS database [15]. Except for *B3gnt5*, all B cell signature genes were up-regulated in infected lungs after day 5 p.i. and continued to be highly expressed until day 22 after which the expression decreased gradually (Figure 5A). Moreover, in immune-histochemical staining, we could confirm the presence of B220-positive cells in newly forming bronchus-associated lymphoid tissue (BALT) at days 5, 10, 14 p.i. (Figure 5C), and also the presence of plasma cells on day 14 within the BALT (data not shown). We also followed the expression of genes that were previously described to be associated with the formation of secondary and primary lymphoid organs [16]. As shown in Figure 5B, the expression of *Cxcl13, Ccl19* and *Tnf* preceded the up-regulation of the B cell specific genes *Cd19* and *Cd79b* suggesting that these genes may encode signaling molecules that are important for the induction of BALT.

In summary, our analysis associated the up-regulation of B cell expression signature genes in the lung transcriptome with the infiltration of B cells and the formation of BALT. In addition, the prior expression of certain chemokines suggests that they may play a role in the induction of BALT formation.

### Expression of Late Host Response Genes Indicates Lung Repair Processes

Many genes were still differentially expressed compared to mock-infected controls (Figure 1C, Table S1) at 30 days and later after infection. GO analysis of up-regulated genes for each day revealed a significant over-representation of *e.g.* tissue development, epithelium development, response to wounding, epithelial cell differentiation, organ development, tissue morphogenesis at the late host response phase (Table S3). These observations indicated that after the acute early and intermediate phase of the host response to an influenza infection, tissue repair processes are still ongoing late after infection and they may even result in permanent changes of the gene expression profiles in the lung. To verify that gene expression changes are not simply due to aging or other environmental effects occurring between day 1 and 60, we compared the lung transcriptome of influenza-infected with mock-infected mice. The latter were treated with buffer and lungs were prepared at days 0, 1, 2, 4, 8, 14 and 60 after treatment. The corresponding PCA clearly shows that infected animals exhibited unique changes after 60 days p.i. that are distinct from day 60 mock-infected mice (Figure S2). Indeed, all mock-infected mice from all time points after treatment grouped together (Figure S2).

### Transcriptome Analysis of Infected Rag2 Knock-out Mice Highlights a Deficiency in the Adaptive Immune Response

**Figure 6:**
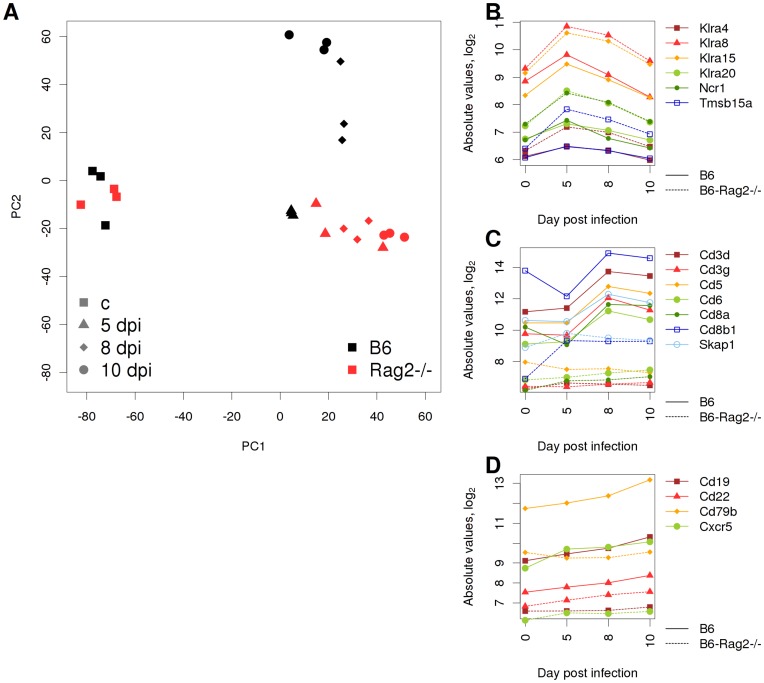
Deficiency in the adaptive immune response of *Rag2* knock-out mice monitored by analysis of genome-wide whole lung gene expression data. (A) A PCA was conducted on all pre-processed probe data of samples from wild type and *Rag2* mutant mice and single replicates were plotted with reference to the first two principal components (PC1, PC2). (B) Gene expression changes of NK cell signature genes, (C) of T cell signature genes and (D) of B cell signature genes in wild type (solid lines) and *Rag2* mutant (dashed lines) are depicted. T and B cell marker genes were selected exhibiting a rank correlation coefficient of ρ>0.95 to Cd8b1 and Cd19 expression in the long-term study, respectively. Abbreviated gene names: killer cell lectin-like receptor, subfamily A, member 4 (*Klra4*); killer cell lectin-like receptor, subfamily A, member 8 (*Klra8*); killer cell lectin-like receptor, subfamily A, member 15 (*Klra15*); natural cytotoxicity triggering receptor 1 (*Ncr1*); thymosin beta 15a (*Tmsb15a*); CD3 antigen, delta polypeptide (*Cd3d*); CD3 antigen, gamma polypeptide (*Cd3g*); CD5 antigen (*Cd5*); CD6 antigen (*Cd6*); CD8 antigen, alpha chain (*Cd8a*); CD8 antigen, beta chain 1 (*Cd8b1*); src family associated phosphoprotein 1 (*Skap1*); CD19 antigen (*Cd19*); CD22 antigen (*Cd22*); CD79B antigen (*Cd79b*); chemokine (C-X-C motif) receptor 5 (*Cxcr5*).

To test if a comparative lung transcriptome analysis will be able to detect defects at the cellular level, we compared altered host responses in mutant and wild type mice. As a proof of principle, we studied the gene expression in *Rag2* knock-out mice. *Rag2* ^−/−^ mice are deficient in the generation of T and B cells, since *Rag2* is essential for the V(D)J rearrangement and deficiency in this molecule stops the maturation step from pro-B/T cells to pre-B/T cells [17], [18], [19]. The global gene expression profiles in the PCA plot showed that until day 5 p.i., wild type and mutant transcriptome changes differed from mock-infected mice but were similar between mutant and wild type (Figure 6A). However, at day 8 and 10 p.i., the wild type transcriptome diverged from the day 5 profile whereas the *Rag2* ^−/−^ showed little changes (Figure 6A). Furthermore, T and B cell signature genes that were found to be up-regulated in wild type mice at days 8 and 10 did not increase in *Rag2* ^−/−^ mice (Figure 6C, D). In contrast, up-regulation of NK cell signature genes was detectable and some genes even increased in mutant mice (Figure 6B).

Thus, the comparison of mutant and wild type mice transcriptome changes in the lung could demonstrate a competent innate immune response and the deficiency in T and B cells in *Rag2* ^−/−^ mice.

## Discussion

**Figure 7:**
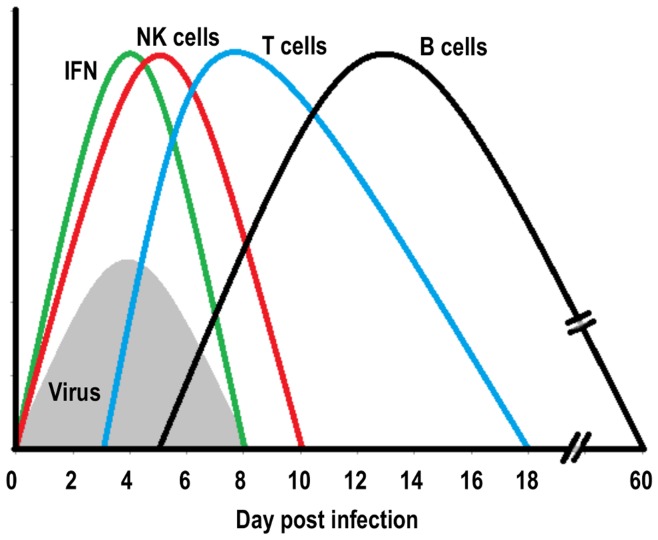
Schematic representation of the activation of distinct immune responses in the host lung during an influenza infection. Increase and decline of viral loads in the infected lungs coincided with the peak of the IFN response. Distinct temporal activation phases can be seen for NK, T and B cell responses. The various waves correspond to schemes shown by [20], but in addition, a B cell response and a shift in the NK cell response has to be noted for the model of influenza A infection.

In this study, we performed a systematic large scale analysis of the global gene expression changes in mouse lungs after influenza A infection over a period of 60 days to describe the systems dynamics at the molecular level and to relate these changes to the host response at the cellular level. Our analysis represents the first comprehensive description of the host response over the entire time course of an infection. The results do not only reveal the activation of the early host response but also include its decline, as well as the stimulation and decrease of the adaptive T cell, and subsequent B cell response (Figure 7). They substantiate the schematic descriptions of the host response which can be found in the textbook [20] with discrete gene expression profiles from NK, T and B cells and type I interferon expression.

In addition to global analyses, like principal component, cluster and signal pathway analysis, we used cell-specific signature genes to follow the activation and infiltration of NK as one important branch of the innate immune response and of T and B cells representing the adaptive immune response. The analysis was performed on whole lungs rather than individual cell populations to visualize all aspects and phases of the host response into a temporal context.

Genome-wide gene expression analyses have been performed previously by several laboratories after infecting cell cultures, mice or other animal models with low or high virulent influenza A virus subtypes [21], [22], [23], [24], [25], [26], [27], [28], [29], [30], [31], [32], [33], [34], [35], [36], [37], [38]. Several publications compared gene expression changes in mouse strains that exhibit differences in susceptibility to infections [22], [27], [28], [38]. These studies revealed important differences in the expression of single genes or genes belonging to particular functional groups. However, all previous analyses were limited to observations for only a short time interval, usually the first 3–5 days after infection. Thus, they did not aim at an integrative global analysis and therefore did not follow the activation, infiltration as well as the decline of individual immune cell populations.

In addition to a global gene expression analysis we also used cell-specific gene signature genes to follow the kinetics and activation of three main immune cell populations, NK, T and B cells. The up-regulation of the NK cell signature genes started in the early response phase on day 3 and reached its peak on day 5 p.i. This response, as well as the expression of interferons coincided with the increase in viral titers shortly after infection [14], [39]. It should be noted that the SPIA indicated a peak of NK cell cytotoxicity at day 8 whereas the peak of NK cell signature gene expression was at day. Here, it is important to know that the canonical KEGG pathway for NK cell cytotoxicity shows considerable overlap with the T cell activation pathway and therefore, the two pathways are not specific for one or the other cell population. In contrast, our signature gene analysis correctly tracked the NK cell response revealing a clear distinction to the infiltration of T cells. This also became evident in the analysis of *Rag2* mutant mice in which the NK cell signature genes indicated NK cell stimulation but T cell signature genes displayed a missing T cell response.

The infiltration of adaptive immune cells was well discernible both by cluster analysis with subsequent GO analysis and by following the expression of T and B cell specific signature genes. The T cell infiltration period was confirmed by flow cytometric analysis and the presence of B cell was validated by histology. Similar kinetics of T cell infiltration was described in experiments on non-lethal infections in mice [40]. At this intermediate phase of infection when the signals for the adaptive immune response increased, viral loads started to decline [14], [39]. These observations are in accordance with many previous reports that correlate the onset of cellular immunity and the production of neutralizing antibodies with clearance of the virus by the host.

The decline in the activation of B cell receptor signaling pathway corresponded to the elimination of viral antigens and may therefore reflect a diminished immune response due to decreasing antigen presentation. The increase in B cell signature genes and pathways indicated the formation of newly formed peripheral lymphoid tissue, bronchus-associated lymphoid tissue (BALT). The formation of BALT is fundamental for clearing an influenza infection [41]. BALTs have distinct B cell follicles and T cell areas and support proliferation of both cell types. Its formation after an influenza infection may follow similar mechanisms as for the generation of other secondary lymphoid organs (lymph nodes, mucosa associated lymphoid tissue in the gut). During the latter, the expression of specific genes in non-lymphoid tissues is thought to induce the supporting lymph node tissues and attract leukocyte precursors [16].

The description of the different steps in the host response to an acute lung infection will form an essential basis to study specific defects in mutant or compromised hosts. To demonstrate the value of our data sets for such an approach, we performed a comparative analysis with *Rag2* knock-out mice. We showed previously that these mice died late after infection, suggesting a normal innate immune response and a deficient adaptive immune response which eventually leads to death [42]. In our comparative transcriptome analysis, the absence of stimulated B and T cells becomes readily apparent because the respective signature genes were not found to be up-regulated. The expression of NK cell signature genes was at a higher level in *Rag2* knock-out mice compared to C57BL/6J controls indicating that the activation of the adaptive immune responses with the corresponding reduction of viral load in wild type mice limits NK cell activation.

The comprehensive data set combined with global bioinformatics analysis as well as with immune cell specific signature genes which we generated will now permit to perform the reconstruction of gene interaction networks. We already performed a first analysis of the transcriptome changes at early time points after infection [43]. Further studies are ongoing including later time points and cell-specific gene signatures.

In conclusion, our results demonstrated that various phases of the host immune response to influenza virus infection can be traced at the transcriptional level. The activation of the innate immune system, the switch from the innate to the adaptive immune response and the establishment of immunity were clearly discernible in the kinetics of gene expression patterns. The comparison to *Rag2* knock-out mice confirmed that our data set is well suitable as a basis to compare normal and altered host responses and provide valuable information about the deficiencies in abnormal hosts.

## Methods

### Ethics Statement

All experiments in mice were approved by an external committee according to the national guidelines of the animal welfare law in Germany (‘Tierschutzgesetz in der Fassung der Bekanntmachung vom 18. Mai 2006 (BGBl. I S. 1206, 1313), das zuletzt durch Artikel 20 des Gesetzes vom 9. Dezember 2010 (BGBl. I S. 1934) geändert worden ist.’). The protocol used in these experiments has been reviewed by an ethics committee and approved by the ‘Niedersächsiches Landesamt für Verbraucherschutz und Lebensmittelsicherheit, Oldenburg, Germany’ (Permit Number: 33.9.42502-04-051/09).

### Virus, Mouse Strains, and Infection

The mouse-adapted virus strain influenza A/Puerto Rico/8/34 (H1N1; PR8; lvPR8/Mun2/EMG/0209) was produced in the allantoic cavity of 10-day-old embryonated hen eggs for 48 h at 37°C. Original stocks of the PR8 virus (Munster variant, see [39] for details) were received from the strain collection at the Institute of Molecular Virology, Muenster, Germany. C57BL/6J mice were obtained from Janvier, France. The generation of *Rag2* mutant mice was described previously [18]. *Rag2* mutant mice were on a C57BL/6J background. Female, 10–12 weeks old mice were anesthetized by intra-peritoneal injection with Ketamine/Xylazine (85% NaCl (0.9%), 10% Ketamine, 5% Xylazine) with doses adjusted to the individual body weight. Mice were then intra-nasally infected with a dose of 2×10^3^ FFU PR8 virus or mock-infected with PBS for controls as described previously [28]. At day 1, 2, 3, 5, 8, 14, 18, 22, 26, 30, 40, 60 post infection (p.i.) mice were sacrificed, each entire lung extracted and stored separately in RNAlater solution (Qiagen) at −80°C. Mice were infected in three independent rounds covering the time spans of 1–5, 1–14 and 1–60 days, respectively. For every day p.i. at least three mice were prepared as three independent biological replicates. For some days samples were repeatedly taken, *e.g.* controls were collected in all rounds and thus all controls add up to nine biological replicates. The comparison of *Rag2* ^−/−^ with C57BL/6J wild type mice was performed accordingly with three replicates for each strain on day 5, 8 and 10 plus non-infected controls. We confirmed that mice were infected by quantitative RT-PCRs for elevated transcript levels of *Il6, Cxcl10*, and *Ccl2* (for the early times) and by monitoring weight loss for later times. Weight loss curves reflected our previously described curves [14], [44]. All mice were maintained under specific pathogen free conditions.

### RNA Extraction and Microarray Experiment

Isolation of RNA from total lungs was performed as described previously [28]. For DNA microarray hybridization and analysis, the quality and integrity of the total RNA was controlled on a 2100 Bioanalyzer (Agilent Technologies; Waldbronn, Germany). 500 ng of total RNA were applied for Cy3-labelling reaction using the one color Quick Amp Labeling protocol (Agilent Technologies; Waldbronn, Germany). Labeled cRNA was hybridized to Agilent’s mouse 4×44 k microarrays for 16 h at 68°C and scanned using the Agilent DNA Microarray Scanner. Expression values were calculated by the software package Feature Extraction 10.5.1.1 (Agilent Technologies; Waldbronn, Germany). The expression of *Ccl2*, *Cxcl10*, and *Il6* was also analyzed by quantitative RT-PCR analysis and they were consistent with the results from the expression arrays (data not shown). The complete data set has been deposited at the ArrayExpress database under the accession number E-MTAB-764.

### Data Analysis

Data were analyzed using the R software, several packages from BioConductor including the packages Agi4×44PreProcess, affycoretools, annotate, RankProd, GOstats, SPIA, KEGGSOAP, Cairo, psych, gplots, RColorBrewer, mgug4122a.db, and gtools. Preprocessing steps included background correction (“normexp”), quantile normalization, probe summarization, and log_2_ transformation. For background correction, fitted intensities were calculated by the convolution of normal and exponential distributions to the observed foreground and background intensities [45]. For a robust analysis, median values were calculated for each gene of 3–9 replicates. We then identified all genes that were differentially expressed (DE) genes. DE genes were defined as genes that exhibited at least a two-fold change in expression levels compared to the controls and the fold-changes had to be significant with an FDR corrected p-value of 0.1 using the rank product method [46], [47]. Cluster analysis (density clustering, which eliminates non-associated genes; data normalized to the mean and standard deviation, Euclidean distance derived by transformation of the Pearson correlation coefficient [48], [49]) was used to find genes with similar expression profiles. In order to further functionally characterize DE genes, GOstats was applied to determine the over-representation of gene ontology terms [50] and SPIA, the signaling pathway impact analysis, to examine KEGG pathways [51]. NK cell signature genes were derived from the transcriptomes of sorted NK cells compared to T cells and whole lungs (using a threshold of differential NK cell expression of 2^0.5^ fold, data not published).

### Preparation of Cell Suspensions and Flow Cytometric Analysis

Mice were euthanized by CO_2_ asphyxiation and lungs were homogenized through a 40 µm nylon (BD Pharmingen, Heidelberg, Germany) mesh. Leukocytes were enriched and erythrocytes and lung tissue removed via density gradient centrifugation (Lympholyte M, Cedarlane, Ontario, CN) followed by washing with PBS/3%FCS. After pre-incubation with 2.4G2 mAb cells were incubated for 20 min at 4°C in the dark with the respective monoclonal antibodies (mAbs). Controls of medium and isotypes were performed simultaneously. After washing, cells were analyzed in a multi-color flow cytometer (FACSCalibur, Becton Dickinson, Heidelberg, Germany) running Cell Quest Pro software. FACS data were analyzed using FlowJo 7.6.1 software (Tree Star, Inc.,Ashland, Oregon, USA). CD16/32 mAb (2.4G2) (eBioscience, San Diego, CA, USA) was used to block FcγRII/III receptor-mediated unspecific binding. For specific attaining, the following mAbs were used: anti-CD3 (145-2C11) AlexaFlour 647 (eBioscience), anti-CD4 (RM4–5) PerCP, anti-CD8 (53-6.7) FITC, PE (BD Pharmingen, Heidelberg, Germany), Isotype-matched mAbs were used as negative controls.

### Histological Analysis

Lungs were extracted from mice *in toto* on indicated days after infection and immersion-fixed for 24–72 h in 4% buffered formaldehyde solution (pH 7.4), dehydrated in a series of graded alcohols and embedded in paraffin. Sections (0.5 µm) were cut with the microtome (Microm HM340E, Thermo Scientific, Walldorf, Germany). Three sections from three different levels of the lungs were stained overnight with the primary biotinylated antibody CD45R/B220 (BD Pharmingen, Heidelberg, Germany) at 4°C. Sections were incubated for 30 min with the secondary antibody (rabbit anti-goat-biotin from KPL, MA, USA). The primary and secondary antibodies were diluted 1∶800 and 1∶250 respectively. Finally, the sections were slightly counterstained with haematoxylin.

## Supporting Information

Figure S1 Activation of innate and adaptive immune response pathways in the lungs of influenza infected C57BL/6J mice. The SPIA algorithm was applied for all DE genes in PR8 infected lungs for different KEGG pathways. Selected pathways of the innate and adaptive immune system are depicted which exhibited a significant change over the time period studied. As criterion for pathway activation, a total net accumulated perturbation of an FDR-corrected p-value of <0.05 was chosen. Please note that no data points are shown for days p.i. that do not exhibit a significant change in the respective pathway. (A) RIG-I receptor signaling pathway, (B) Cytokine-cytokine receptor interaction, (C) Chemokine signaling pathway, (D) Natural Killer cell mediated cytotoxicity pathway, (E) T cell receptor signaling pathway, (F) B cell receptor signaling pathway.
(TIFF)

Figure S2 Gene expression of mock-infected mice is similar at different days after treatment but different to infected mice. The Principle Component Analysis (PCA) of mock-infected and infected mouse lung transcriptomes taken over the investigated time interval reveals the grouping together of all mock-infected samples compared to infected samples. Single replicates were plotted with reference to the first two principal components (PC1, PC2) covering 54.3% of the total variance. Symbols indicate biological replicates prepared at the same day p.i. from different individuals. The red and black colors represent infected and mock-infected mice, respectively.
(TIF)

Table S1 Gene expression values from all genes described in Figure 2. The information on cluster number, gene symbols, Entrez gene identifier, fold changes over time as log_2_ values, and descriptions of genes is listed. NA: missing values, gene not differentially expressed at the corresponding day after infection.
(XLS)

Table S2 GO term analysis for all DE cluster genes depicted in Figure2. GOBPID: GO term identifier; Pvalue: hypergeometric test; ExpCount: expected count, number of genes expected if randomly selected; Count: observed number of genes; size: number of all genes detected on the microarray chip associated with the GO term; Term: description of the GO term; cluster: number of cluster as described in Figure 2.
(XLS)

Table S3 GO term analysis of all DE genes per day for each day p.i. GOBPID: GO term identifier; Pvalue: hypergeometric test; ExpCount: expected count, number of genes expected if randomly selected; Count: observed number of genes; size: number of all genes detected on the microarray chip associated with the GO term; Term: description of the GO term; day: day post infection.
(XLS)
