# Supplementary figures and images for: Global Transcriptome Analysis in Influenza-Infected Mouse Lungs Reveals the Kinetics of Innate and Adaptive Host Immune Responses
Source: PLoS One. 2012 Jul 17;7(7):e41169. doi: 10.1371/journal.pone.0041169 (PMC3398930; doi:10.1371/journal.pone.0041169)

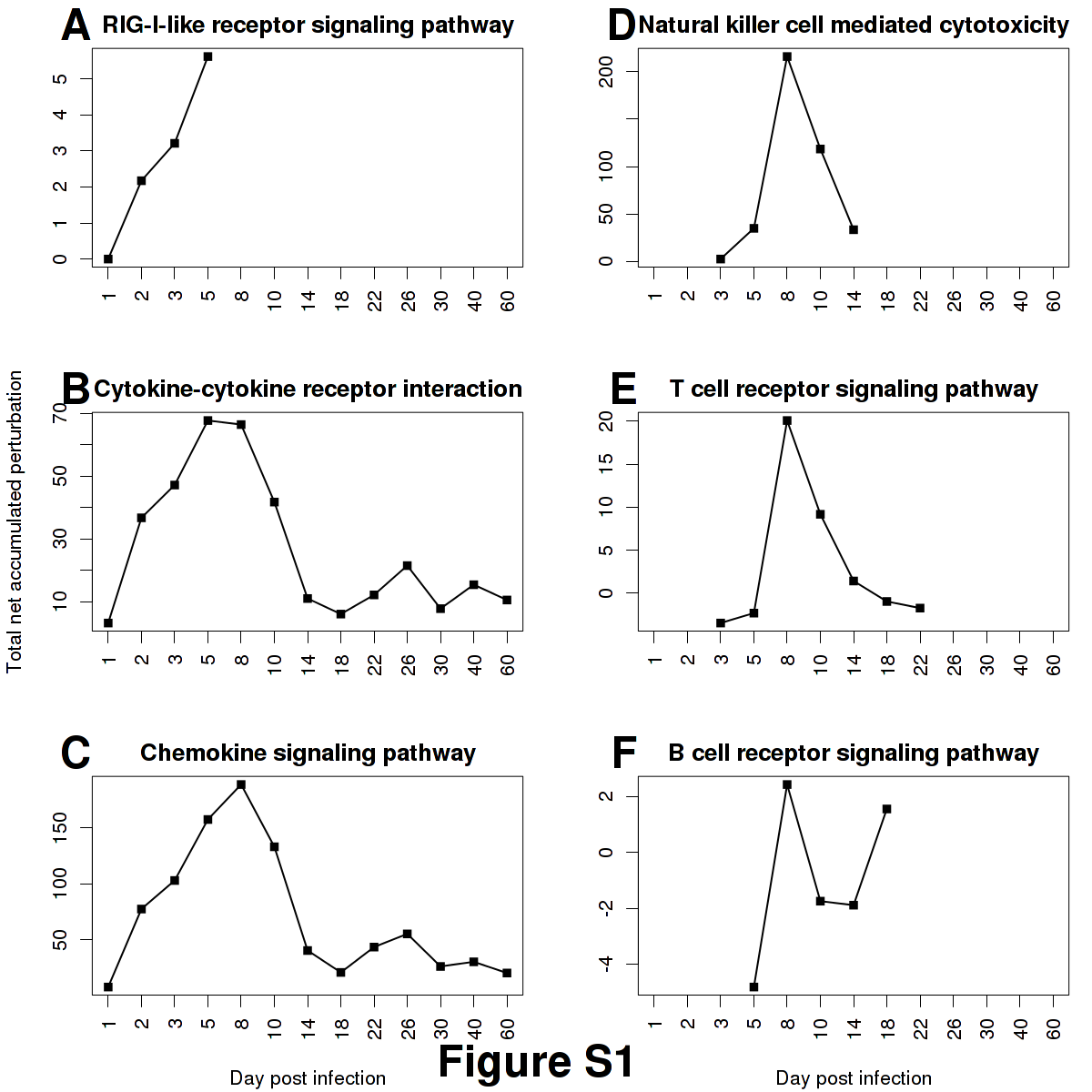

Supplement: Figure S1 — Activation of innate and adaptive immune response pathways in the lungs of influenza infected C57BL/6J mice. The SPIA algorithm was applied for all DE genes in PR8 infected lungs for different KEGG pathways. Selected pathways of the innate and adaptive immune system are depicted which exhibited a significant change over the time period studied. As criterion for pathway activation, a total net accumulated perturbation of an FDR-corrected p-value of <0.05 was chosen. Please note that no data points are shown for days p.i. that do not exhibit a significant change in the respective pathway. (A) RIG-I receptor signaling pathway, (B) Cytokine-cytokine receptor interaction, (C) Chemokine signaling pathway, (D) Natural Killer cell mediated cytotoxicity pathway, (E) T cell receptor signaling pathway, (F) B cell receptor signaling pathway. (TIFF) [file pone.0041169.s001.tif]

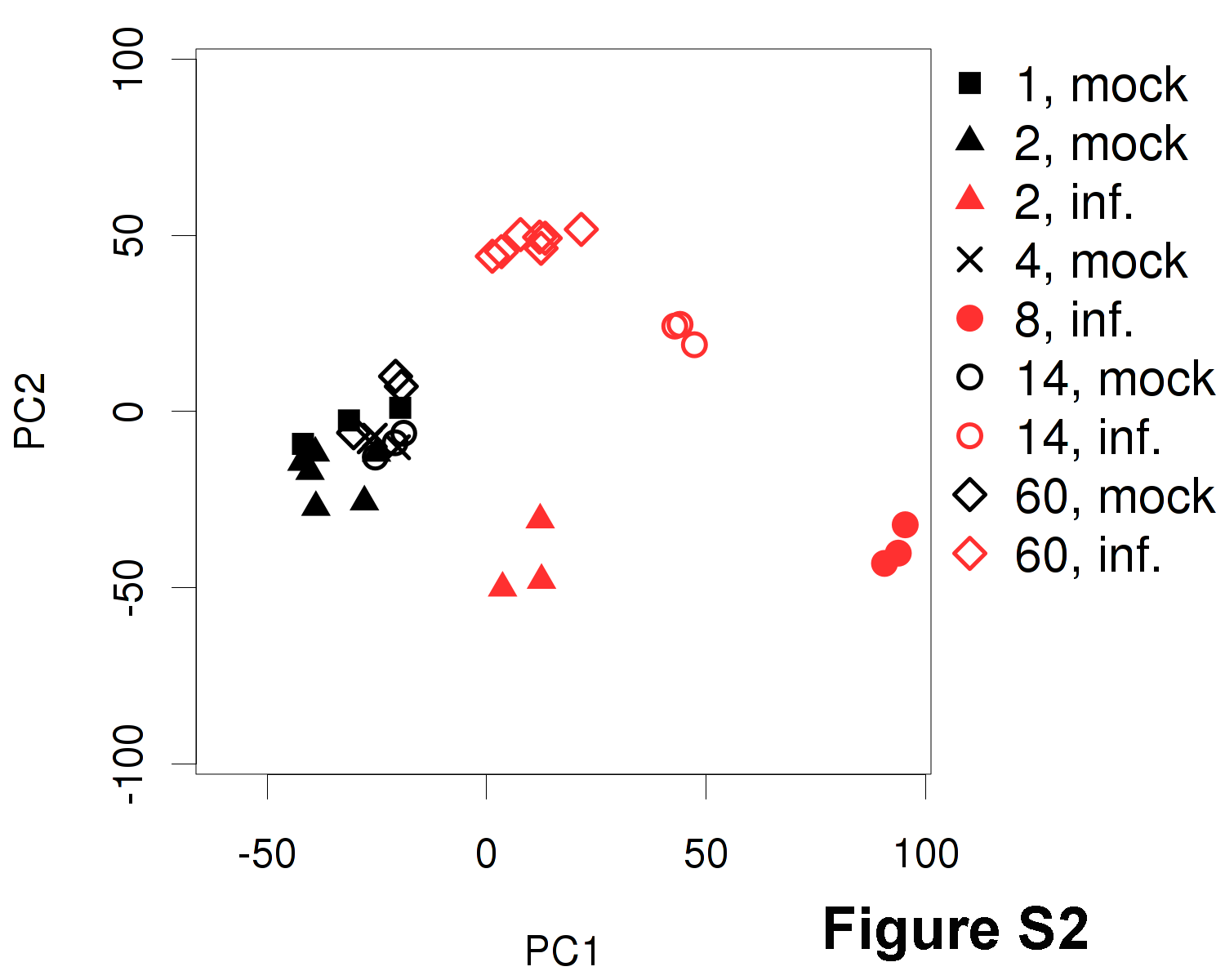

Supplement: Figure S2 — Gene expression of mock-infected mice is similar at different days after treatment but different to infected mice. The Principle Component Analysis (PCA) of mock-infected and infected mouse lung transcriptomes taken over the investigated time interval reveals the grouping together of all mock-infected samples compared to infected samples. Single replicates were plotted with reference to the first two principal components (PC1, PC2) covering 54.3% of the total variance. Symbols indicate biological replicates prepared at the same day p.i. from different individuals. The red and black colors represent infected and mock-infected mice, respectively. (TIF) [file pone.0041169.s002.tif]
